# Effectiveness of an educational video in older adults’ perception about falling risks: a randomized clinical trial*

**DOI:** 10.1590/1980-220X-REEUSP-2021-0417

**Published:** 2022-04-13

**Authors:** Guilherme Guarino de Moura Sá, Ana Maria Ribeiro dos Santos, Khelyane Mesquita de Carvalho, Nelson Miguel Galindo, Márcia Teles de Oliveira Gouveia, Elaine Maria Leite Rangel Andrade

**Affiliations:** 1 Instituto Federal de Educação, Ciência e Tecnologia de Pernambuco, Departamento de Enfermagem, Belo Jardim, PE, Brazil.; 2 Universidade Federal do Piauí, Programa de Pós-Graduação em Enfermagem, Teresina, PI, Brazil.; 3 Universidade Federal do Piauí, Colégio Técnico de Teresina, Teresina, PI, Brazil.; 4 Instituto Federal de Educação, Ciência e Tecnologia de Pernambuco, Departamento de Enfermagem, Pesqueira, PE, Brazil.

**Keywords:** Aged, Accidental Falls, Health Education, Instructional Film and Video, Geriatric Nursing, Clinical Trial, Anciano, Accidentes por Caídas, Educación em Salud, Película y Video Educativos, Enfermería Geriátrica, Ensayo Clínico, Idoso, Acidentes por Quedas, Educação em Saúde, Filme e Vídeo Educativo, Enfermagem Geriátrica, Ensaio Clínico

## Abstract

**Objective:**

to assess the effectiveness of using an educational video in comparison with verbal nursing guidelines in increasing older adults’ perception of falling risks.

**Method:**

this is a randomized clinical trial in a cluster, with 138 community older adults, randomized into an intervention group, which watched an educational video, and a control group, which received verbal instructions. The perception of falling risks was assessed by FRAQ-Brazil in the pre-test and after a 30-day follow-up. Student’s t-test was used for dependent samples to compare intragroup means and for independent samples to compare intergroup means. The effect size was determined by Cohen’s d.

**Results:**

in the intragroup analysis, intervention and control groups had an increase in perception, with a statistically significant difference between pre-and post-tests. In the intergroup analysis, the control group showed a greater increase in the perception of falling risks in relation to the intervention group (p = 0.013), with Cohen’s d of small effect.

**Conclusion:**

the use of an educational video and verbal instructions increased older adults’ perception of falling risks, with better results in the control group. However, the effect size was small. RBR-8nfggd.

## RESUMO

**Objetivo:**

avaliar a efetividade do uso de vídeo educativo, em comparação com orientações verbais de enfermagem no aumento da percepção de idosos sobre riscos de queda.

**Método:**

ensaio clínico randomizado em *cluster*, com 138 idosos na comunidade, aleatorizados em grupo intervenção, que assistiu vídeo educativo, e grupo controle, que recebeu orientações verbais. A percepção dos riscos de queda foi avaliada pelo FRAQ-Brasil no pré-teste e após seguimento de 30 dias. Utilizou-se Teste t de Student para amostras dependentes, para comparação das médias intragrupo, e Teste t de Student, para amostras independentes, para comparação de médias intergrupos. O tamanho do efeito foi determinado pelo d de Cohen.

**Resultados:**

na análise intragrupo, os grupos intervenção e controle tiveram aumento da percepção, com diferença estatisticamente significativa entre pré e pós-teste. Na análise intergrupo, o grupo controle apresentou maior aumento da percepção dos riscos de queda em relação ao grupo intervenção (p = 0,013), com d de Cohen de efeito pequeno.

**Conclusáo:**

o uso do vídeo educativo e as orientações verbais aumentaram a percepção de idosos sobre riscos de queda, com melhores resultados no grupo controle. Contudo, o tamanho do efeito foi pequeno. RBR-8nfggd.

## RESUMEN

**Objetivo:**

evaluar la efectividad del uso de un video educativo, frente a orientaciones verbales de enfermería, en el aumento de la percepción de los ancianos sobre el riesgo de caídas.

**Método:**

ensayo clínico aleatorizado en conglomerado, con 138 ancianos de la comunidad, aleatorizados en un grupo intervención, que vio un video educativo, y un grupo control, que recibió instrucciones verbales. La percepción del riesgo de caída fue evaluada por FRAQ-Brasil en la prueba previa y después de un seguimiento de 30 días. Se utilizó la prueba t de Student para muestras dependientes para comparar medias intragrupos y la prueba t de Student para muestras independientes para comparar medias intergrupos. El tamaño del efecto se determinó mediante la d de Cohen.

**Resultados:**

en el análisis intragrupo, los grupos de intervención y control tuvieron un aumento en la percepción, con una diferencia estadísticamente significativa entre la prueba previa y posterior. En el análisis intergrupal, el grupo control mostró mayor aumento en la percepción de los riesgos de caída en relación al grupo intervención (p = 0,013), con d de Cohen de efecto pequeño.

**Conclusión:**

el uso de video educativo e instrucciones verbales aumentó la percepción de los ancianos sobre el riesgo de caídas, con mejores resultados en el grupo control. Sin embargo, el tamaño del efecto fue pequeño. RBR-8nfggd.

## INTRODUCTION

Falls are an important external cause of morbidity and mortality in older adults population worldwide, and fall ­prevention is one of the main challenges among health professionals and researchers in the areas of gerontology and geriatrics^(1–2)^. These accidents are a result of a synergistic interaction of biological, socioeconomic, environmental and behavioral factors^(3)^. Thus, preventive interventions should consider the multifactorial nature of this problem.

The World Health Organization (WHO) model for fall prevention in old age proposes an action plan, which points out the importance of increasing awareness about the prevention of these accidents, improving risk assessment and implementing interventions^(3)^. Thus, strategies to cope with falls in older adults should promote the empowerment of this population, in order to increase the perception of the risks to which they are exposed^(4)^.

The perception of risk can be understood as the interpretation or understanding of a person at each dangerous event or specific threat^(5)^. Regarding older adults’ perception about falling risks, studies conducted in Australia and Brazil revealed that most of this population underestimates or does not recognize their vulnerability to this accident^(6–7)^. In this context, nurses have a strategic role in preventing falls and increasing this perception in older adults, as they are inserted in the various levels of geriatric care, acting in awareness and behavior change, especially through health education, employed especially in verbal guidelines in Primary Health Care (PHC)^(8)^. In the field of health education, the technical-scientific advancement provided the advent of educational technologies, which have been incorporated into nursing performance as teaching tools in health^(9–10)^.

Among the educational technologies for community older adults, identified in an integrative review, the educational video emerged as a tool that promoted improvement of different outcomes in experiments with this audience. Moreover, its use was effective in different aspects related to falls, such as identification and reduction of risks, level of motivation for self-care and knowledge to prevent dangerous behaviors^(11)^. Thus, the educational video is presented as a technological instrument, which allows using simultaneous and playful resources, providing the standardization of instructions and conveying of information to a greater number of people at the same time^(12–13)^.

Therefore, it was established as a hypothesis that the use of educational video is effective in increasing older adults’ perception about falling risks, compared to verbal nursing guidelines. However, although this technology is presented as a resource to provide the health education process with the necessary tools, there is no evidence in the literature about its effects on older adults’ perception about falling risks in the Brazilian reality. Therefore, it is necessary to provide evidence that addresses this knowledge gap, with a view to contributing to providing the health education process with older adults in nurses’ practice with the necessary tools, especially in PHC. From this, this study aimed to assess the effectiveness of using an educational video compared to verbal nursing guidelines in increasing older adults’ perception about falling risks.

## METHOD

### Design of Study

This is a randomized clustered controlled clinical trial with two parallel groups, with 1:1 allocation rate, conducted from May to September 2019. For the study report, the Consolidated of Reporting Trials (consort) for Randomized Trials of Nonpharmacological Treatments^(14)^ was used.

### Population

The population consisted of 1,773 older adults aged 65 years and older, registered in the urban area of PHC, in the city of Bom Jesus, PI, Brazil.

### Local

The data collection site was the Basic Health Unit (BHU) of each of the nine Family Health Strategy (FHS) teams of the city urban area.

### Selection Criteria

Participants with age equal to or greater than 65 years, without cognitive impairment, assessed by the Mini Mental State Examination (MMSE), with cut-off points defined from education^(15)^, not presenting physical impossibility of locomotion to the BHU were included. Participants planning to move to another city before the data collection completion period, presenting hearing, visual or speech impairments – these conditions were verified through the information obtained from the unit’s Community Health Worker (CHW) and nurse – were excluded. People could be discontinued from the study if they did not return to the BHU or were not located for post-test assessment.

### Sample Definition

The sample size definition was based on the equation for comparison between two groups, 95% confidence level, 80% test power and 25% expected clinical difference, based on a ­previous study^(7)^. The calculations indicated a minimum sample of 56 older adults in each group, totaling 112. When considering the possible losses, 50% of this total was added, so it was necessary to recruit at least 168 older adults.

### Data Collection

The primary outcome of interest was the mean perceived risk of falling, and the secondary outcome was the ­percentage of correct answers for the questionnaire items. For data collection, two instruments were used: the Fall Risk Perception Questionnaire (FRAQ-Brazil)^(4)^ and a script to characterize demographic, clinical and fall data.

The FRAQ-Brazil was used to assess the outcomes of this study. This instrument was developed by Canadian researchers and presented construct validity and reasonable test-retest reliability. In the present study, the FRAQ-Brazil was used, which has semantic, idiomatic, cultural and conceptual ­equivalences for older adults aged 65 years and over, internal consistency, with Cronbach’s Alpha of 0.95, intra-examiner equivalence with a Kappa coefficient of 0.89 and inter-examiner of 0.78. The instrument is divided into two parts: the first (part A) has two open-ended questions, which investigate the prior knowledge of older adults about the causes of falls and how they obtained this information, and a closed-ended question, on the opinion of older adults regarding the possibility of being susceptible to fall at any time; the second (part B) consists of 25 multiple-choice questions about fall risks. The final score is obtained from the sum of the number of correct answers, indicated in the questions in part B. All questions had only one correct alternative. However, a question contained eight correct answers and for each correct answer a point is assigned, so that the FRAQ-Brazil score varies from zero to 32 and the greater the number of points, the better the perception of fall risks^(4)^.

A script was prepared by the members of the Study Group on Aging and External Causes of Morbidity and Mortality (GEECEM – *Grupo de Estudos em Envelhecimento e Causas Externas de Morbimortalidade*) of the *Universidade Federal do Piauí* (UFPI) and submitted to validation by five judges, experts in gerontology and geriatrics. An adapted version with questions was used to collect sociodemographic (sex, age, reading and writing, years of study and family composition), economic (family income), clinical (physical exercise) and fall (fall in the last year) data.

Prior to the start of the interventions, participants were randomized into their respective groups. To reduce the risk of sample contamination, through contact between participants in the intervention group (IG) and control group (CG), cluster randomization was chosen, so that the clusters corresponded to the FHS teams and their coverage area. Of the nine health teams, one was previously drawn to conduct the pilot study and was not part of the final sample. Thus, eight teams corresponded to the clusters that were randomized by simple random allocation of 1:1, in parallel groups, of which four teams composed the IG and another four the CG. Randomization was performed using the R by a professional who did not participate in data collection. To define the random sequence, a list of teams was organized, starting in the ascending order of their respective registration numbers in FHS. When considering the numerical sequence generated by R, which defined IG and CG, the teams were allocated. The number of participants in each cluster was defined equitably and proportionally to the number of older adults aged 65 and over registered in each health team.

A pilot study was conducted, from May to June 2019, to test the feasibility of recruiting the sample, the time demand needed to apply the instruments, promote the setting and improve the interventions. Participants were 18 older adults registered in the previously selected FHS team. In this stage, the four micro-areas of the team were the clusters, randomized by simple random allocation into two groups, which constituted the IG and CG, with nine older adults in each. The pilot study followed the entire clinical trial methodological operationalization and its participants did not make up the final study sample. There was no change in data collection procedures after a pilot study. However, the team of pre-test interviewers was expanded for the clinical trial.

The final team of 15 interviewers was formed by nursing professors and nursing students from UFPI. The interviewers were divided into two teams: the first, with nine interviewers, to apply the instruments in the pre-test of IG and CG; the second, with six other interviewers, to apply the questionnaire in the IG and CG post-test. The two teams were trained by the main researcher at different times. The recruitment and follow-up period took place from June to August 2019, with the participants, from IG and CG, organized into subgroups of up to ten older adults. The operationalization of data collection occurred equally in IG and CG, in two stages, with only the difference in the intervention applied.

(1) *First stage:* data collection was scheduled in each BHU and the unit nurse was asked to list potentially eligible older adults in the area covered by the health team, based on the inclusion criteria. Based on this indication, a draw was carried out to define the participants of each team by a professional who did not participate in the data collection. The randomly selected older adults were invited on a home visit by the CHW, attended the BHU and, after accepting to participate in the research, signed the Informed Consent Form (ICF). In cases of illiteracy, the ICF was read to older adults and a witness, and the fingerprint of participants’ thumb was collected.

Then, in an individual interview in a private place of BHU, MMSE was applied and data were collected for characterization. Moreover, a pre-test assessment of the perception of fall risks was carried out using the FRAQ-Brazil. Soon after, participants were sent to the BHU meeting room for group educational activity. In both the IG and the CG, older adults were accommodated in chairs, arranged equidistantly in a semicircle.

The IG watched an educational video entitled “*Risco de queda: não caia nessa*”, which was constructed based on the Cognitive Theory of Multimedia Learning and selected content based on the WHO fall prevention model and FRAQ-Brazil items. It had a digital animation format, audio narration, duration of ten minutes and five seconds and contemplated biological, ­socioeconomic, environmental and behavioral risks of falls in older adults. The video was validated by nurses with expertise in geriatrics, gerontology and falls and assessed by older adults^(16)^. The intervention was conducted by a nurse, who did not compose the team of interviewers. The video was projected on a white wall, through a multimedia projector and audio ­transmitted by a speaker with Rms 80w power and a frequency of 100 Hz – KHz, displayed only once, without pause or ­repetition, and no questions were answered, in order not to influence the assessment of study outcomes. At the end, older adults were invited to return 30 days later for post-test assessment.

**Figure 1:**
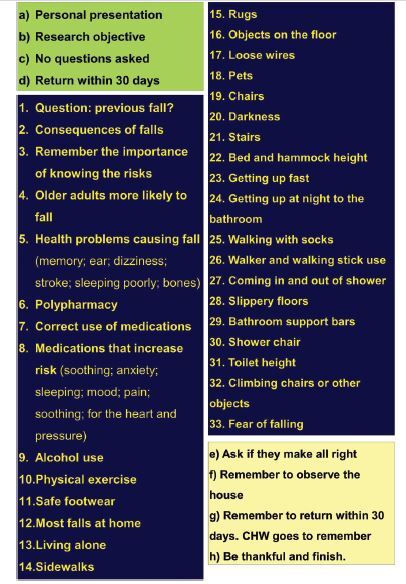
Poster with topics of the Standard Operating Procedure used by a nurse in verbal guidelines for the control group. Bom Jesus, PI, Brazil, 2019.

The CG received verbal guidance on fall risk in older adults by a nurse previously trained by the main researcher and who was not part of the team of interviewers. For this study, a Standard Operating Procedure (SOP) was built to promote the standardization of exposures to all subgroups of ten older adults. The SOP contained a procedure definition, necessary materials, personal presentation, environment organization, and content about falling risks, addressed in the same sequence presented in the video. In order to guide the nurse and ensure standardization of information in all subgroups, a 150 × 90 cm poster was constructed, which contained reminders of all SOP topics (Figure 1).

The poster was fixed on the wall located behind the row of chairs in which older adults were accommodated and in front of a nurse, so that participants could not see it. The nurse positioned herself in the center of the semicircle and did not present any teaching material, such as images or videos. Thus, there was only oral exposure. Exposure time in the subgroups ranged from 25 to 30 minutes. At the end, older adults were also invited to return 30 days later for post-test assessment.

(2) *Second stage:* an interview was conducted for post-test assessment of the perception of fall risks by FRAQ-Brazil, 30 days after the first stage, at the BHU. The CHW ­reinforced the invitation up to two days before the scheduled date. Older adults who did not attend were contacted by telephone to schedule an interview, or received up to three home visit attempts to locate and fill out the instrument.

Blinding was not possible to be applied to the IG and CG older adults, since they knew the intervention to which they were submitted, as well as the pre-test research team and the researchers who conducted the groups. Blinding was applied to the post-test interviewees in both groups, as they did not know the intervention applied to each participant and did not receive information on the procedures previously adopted. Also, throughout the process of tabulation and data processing, the professional responsible for statistical analysis was blinded, through group coding, in G1 and G2, in the database.

### Data Analysis and Treatment

Data were analyzed in the Statistical Package for the Social Sciences, version 21.0. Compliance with the normal distribution of numerical variables was verified by the Kolmogorov-Smirnov test. From the characteristics of each variable, statistical tests were determined. A 5% significance level was considered, and the principles of analysis per protocol were followed. Categorical variables were described as absolute and relative frequencies, and numerical variables, as mean and standard deviation or median and interquartile range. The group homogeneity was tested by applying Student’s t-test for independent samples, Mann-Whitney U test and Chi-square test for proportion.

To compare the proportions of correct answers of FRAQ-Brazil items between groups, the chi-square test for proportion and Fisher’s exact test were adopted. The effect of the interventions was assessed by comparing the means of the final FRAQ-Brazil score of intra-and inter-group participants, using Student’s t-test for dependent and Student’s t-test for independent samples, respectively. The effect size was ­established by Cohen’s d and classified as negligible (<0.19), small (0.20–0.49), medium (0.50–0.79) or large (0.80–1.29)^(17)^.

### Ethical Aspects

The study complied with Resolution 466/12 of the Brazilian National Health Council (*Conselho Nacional de Saúde*). It was approved by the Research Ethics Committee of UFPI in 2019, under Opinion 3.334.943, and registered in the Brazilian Clinical Trials Registry database, with primary identifier: RBR-8nfggd.

## RESULTS

**Figure 2:**
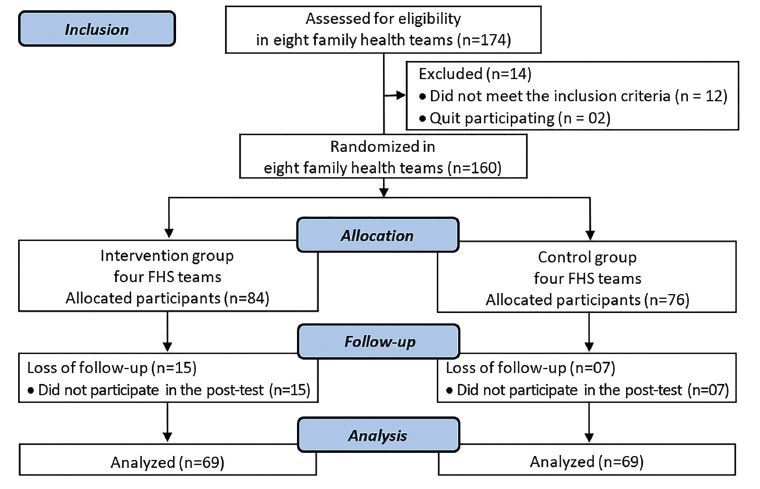
Diagram of participant flow in the study. Bom Jesus, PI, Brazil, 2019.

During the study period, 174 older adults were recruited to assess eligibility. Only 160 met the inclusion criteria and were allocated to the respective groups. In the 30-day follow-up there was a loss of 22 older adults, so 138 completed the study (IG = 69; CG = 69) (Figure 2). The reason for the losses was related to the discontinuity criteria.

Most participants were female (66.7%), aged 65 to 79 years (81.9%), with a mean age of 73.5 years (SD = 6.2), unable to read and write (53.6%), with a median of one year of study (IR = 0–3), median family composition of two people (IR = 1–4) and median monthly family income of 1,996.00 (IR = 998.00–1,996.00), considering the minimum wage of Brazil in 2019 (R$998.00 – about US$184.81). It was found that most older adults practiced physical exercise (55.8%), had not suffered a fall in the last year (61.6%), felt that they were at risk of falling at any time (63.8%) and had not received information about fall risks (83.3%). Those who received these guidelines reported having been guided by CHWs (66.2%), nurses (40.0%), physicians (33.0%), television (30.8%), social worker (10.0%), nursing students (10.0%) and one could not inform which professional (10.0%). IG and CG were homogeneous at baseline, in relation to sociodemographic, clinical and fall variables (p > 0.05) (Table 1).

**Table 1.** Distribution of sociodemographic, economic, clinical and fall characteristics of 138 older adults according to intervention and control groups – Bom Jesus, PI, Brazil, 2019.

Categorical variables	Intervention group (n = 69)	Control group (n = 69)	p
	n (%)	n (%)	
**Sex**			
Male	22 (31.9)	24 (34.8)	0.718*
Female	47 (68.1)	45 (65.2)	
**Age group**						
65 to 79 years	55 (79.7)	58 (84.1)	0.507*
≥80 years	14 (20.3)	11 (15.9)	
**Read and write**						
Yes	32 (46.4)	32 (46.4)	1.000*
No	37 (53.6)	37 (53.6)	
**Physical exercise**						
Yes	37 (53.6)	40 (58.0)	0.607*
No	32 (46.4)	29 (42.0)	
**Fall in the last year**						
Yes	22 (31.9%)	31 (44.9%)	0.115*
No	47 (68.1%)	38 (55.1%)	
**Feel at risk of falling**						
Yes	45 (65.2%)	43 (62.3%)	0.723*
No	24 (34.8%)	26 (37.7%)	
**Received information on fall risks**			
Yes	13 (18.8%)	10 (14.5%)	0.647*
No	56 (81.2%)	59 (85.5%)	
**Numerical variables**	**Mean (SD^+^)**	**Mean (SD^+^)**	**p**
**Age (years)**	73.6 (6.4)	73.5 (6.2)	0.871^‡^
	**Median (IR^§^)**	**Median (IR^§^)**	**p**
**Education (years)**	0 (0–4)	1 (0–3)	0.817^||^
**Family composition**	2 (1–4)	2 (1–3.5)	0.821^||^
**Family income**	1996 (998–1996)	1996 (998–1996)	0.244^||^

*Chi-square test; ^†^SD = standard deviation; ^‡^Student’s t-test for independent samples; ^§^IR = Interquartile range; ^||^Mann-Whitney U test; ^¶^Current minimum wage = R$998.00, Brazil, 2019.

Regarding the FRAQ-Brazil scores in the pre-test, there was no statistically significant difference between IG and CG. There was a statistically significant increase in the mean scores between pre-and post-tests, both in IG and in CG. However, the mean difference between the two moments was higher in CG. The mean of FRAQ-Brazil scores, verified in the post-test in CG, presented a higher value than the mean of IG. Despite the statistically significant differences observed in the comparison between groups, the effect size of using an educational video, in comparison with the verbal nursing guidelines on older adults’ perception about falling risks, from Cohen’s d, was small to be considered clinically important (Table 2). There was no report of participants about damage or unwanted effects from the interventions.

**Table 2.** Intra-group and inter-group comparison of the mean FRAQ-Brazil scores of the 138 study participants and effect size – Bom Jesus, PI, Brazil, 2019.

Group	Pre-test		Post-test		p^‡^	Difference	
	Mean (SD*)	95%^†^ CI	Mean (SD*)	95%^†^ CI		Mean (SD*)	95%^†^ CI
Intervention group	19.2 (3.5)	18.3–20.0	21.7 (2.7)	21.0–22.4	0.001	2.5 (3.6)	1.7–3.4
Control group	18.8 (3.7)	17.9–19.7	22.8 (2.5)	22.2–23.4	<0.001	4.0 (4.3)	3.0–5.0
p^§^	0.559		0.013			0.030	
d^||^	0.10		0.43			0.38	

*SD = standard deviation; ^†^CI = confidence interval; ^‡^Student’s t-test for dependent samples; ^§^Student’s t-test for independent samples; ^||^Cohen’d.

In the analysis of the correct answers of each item in pre-and post-tests of IG and CG, it was found that, in the pre-test, seven items (2,7,10,15,24,27,29) showed a statistically significant difference and there was a similarity of groups in the number of correct answers in 25 items. In the post-test, three items (3,18,29) showed a statistically significant difference, and the groups were similar with regard to the number of correct answers in 29 items, as observed in Table 3.

**Table 3.** Percentage of correct answers between intervention and control groups in the items to assess older adults’ perception about fall risks in pre-and post-tests – Bom Jesus, PI, Brazil, 2019.

Items	Pre-test		p	Post-test		p
	Intervention group	Control group		Intervention group	Control group	
	n (%)	n (%)		n (%)	n (%)	
1. People aged 65 and over are more likely to fall than younger adults	57 (82.6)	64 (92.8)	0.070*	69 (100.0)	66 (95.7)	0.245^†^
2. Older people can change their activities to prevent falls	62 (89.9)	53 (76.8)	0.040*	65 (94.2)	65 (94.2)	1.000^†^
3. Most falls result in no effect	1 (1.4)	0 (0.0)	1.000^†^	3 (4.3)	11 (15.9)	0.024*
4. Falls make older adults less confident to move around	63 (91.3)	63 (91.3)	1.000*	65 (94.2)	69 (100.0)	0.120^†^
5. Falls are more likely to happen at home	34 (49.3)	36 (52.2)	0.733*	44 (63.8)	46 (66.7)	0.721*
6. Older age increases falling risks	65 (94.2)	63 (91.3)	0.511*	66 (95.7)	67 (97.1)	1.000^†^
7. Using a correct walker does not increase the chance of falling	53 (76.8)	38 (55.1)	0.007*	57 (82.6)	62 (89.9)	0.217*
8. The safest type of footwear is tennis	17 (24.6)	24 (34.8)	0.192*	32 (46.4)	31 (44.9)	0.864*
9. There is a higher risk of falling when entering and exiting the shower	34 (49.3)	32 (46.4)	0.733*	36 (52.2)	47 (68.1)	0.056*
10. Lower risk of falling if living with a family	49 (71.0)	59 (85.5)	0.039*	54 (78.3)	61 (88.4)	0.110*
11. Alzheimer’s affects chances of falling	59 (85.5)	58 (84.1)	0.813*	64 (92.8)	67 (97.1)	0.441^†^
12. Brain stroke affects chances of falling	67 (97.1)	64 (92.8)	0.441^†^	69 (100.0)	69 (100.0)	–^§^
13. Deafness increases chances of falling	42 (60.9)	43 (62.3)	0.861*	54 (78.3)	58 (84.1)	0.384*
14. Ear problems affect chances of falling	61 (88.4)	56 (81.2)	0.236*	64 (92.8)	69 (100.0)	0.058*
15. Eating salty fries does not cause falls	28 (40.6)	47 (68.1)	0.001*	34 (49.3)	39 (56.5)	0.495^†^
16. Use of alcohol increases falling risks	69 (100.0)	68 (98.6)	1.000^†^	69 (100.0)	69 (100.0)	–^§^
17. Medications for anxiety worry or stress may increase chances of falling	14 (20.3)	23 (33.3)	0.084*	31 (44.9)	30 (43.5)	0.864*
18. Sleeping pills may increase chances of falling	27 (39.1)	21 (30.4)	0.284*	30 (43.5)	47 (68.1)	0.004*
19. Mood stabilizers may increase chances of falling	13 (18.8)	9 (13.0)	0.352*	21 (30.4)	17 (24.6)	0.446*
20. Tranquillizers that control symptoms such as hallucination can increase chances of falling	12 (17.4)	15 (21.7)	0.520*	19 (27.5)	19 (27.5)	1.000*
21. Blood pressure medications may increase chances of falling	22 (31.9)	19 (27.5)	0.576*	27 (39.1)	24 (34.8)	0.597*
22. Pain killers may increase chances of falling	10 (14.5)	8 (11.6)	0.613*	12 (17.4)	10 (14.5)	0.642*
23. Morphine pain medications may increase chances of falling	17 (24.6)	12 (17.4)	0.296*	22 (31.9)	25 (36.2)	0.590*
24. Heart medications may increase chances of falling	21 (30.4)	11 (15.9)	0.044*	25 (36.2)	22 (31.9)	0.590*
25. Older adults who take several medications have a greater chance of falling than those who take only one medication	49 (71.0)	45 (65.2)	0.465*	57 (82.6)	55 (79.7)	0.663*
26. Staying physically active decreases chances of falling	53 (76.8)	59 (85.5)	0.191*	57 (82.6)	59 (85.5)	0.642*
27. Getting up at night to go to the bathroom can lead to falls	61 (88.4)	50 (72.5)	0.018*	65 (94.2)	65 (94.2)	1.000*
28. Sitting on the edge of the bed for a minute is the best way to get out of bed	64 (92.8)	59 (85.5)	0.171*	68 (98.6)	67 (97.1)	1.000*
29. Women aged 65 and over have a greater chance of falling	16 (23.2)	28 (40.6)	0.028*	28 (40.6)	42 (60.9)	0.027^†^
30. There is a greater chance of being injured when having weak or brittle bones	67 (97.1)	68 (98.6)	1.000^†^	69 (100.0)	68 (98.6)	1.000*
31. Fear of falling increases chances of falling	55 (79.7)	49 (71.0)	0.236*	58 (84.1)	60 (87.0)	0.629*
32. Having an active dog at home contributes to falls	61 (88.4)	54 (78.3)	0.110*	64 (92.8)	69 (100.0)	0.058^†^

*Chi-square test; ^†^Fisher’s exact test; ^§^Impossibility of performing the test due to similarity between groups, which made the analysis tended to zero.

## DISCUSSION

The results of this study showed that both the use of educational video and verbal nursing guidelines promoted an increase in older adults’ perception about falling risks. Although the greatest increase was observed in CG, effect size was small when compared to IG. Thus, the use of this technology should not compete or replace the verbal guidelines of nurses, but rather be incorporated as a strategic resource in a health education program to prevent falls in older adults.

The higher number of correct answers in the FRAQ-Brazil questions observed among CG older adults supports the result of a meta-analysis, which compared the use of technologies, such as videos and software, with direct verbal instructions to the patient and showed that these were preferred by this public^(18)^. An integrative review that investigated the health education process for older adults who experienced falls concluded that the success of this intervention is enhanced by the construction of a bond, through direct assistance between professional and patient^(19)^. In this sense, it is believed that verbal persuasion and personal contact favored the interpersonal relationship between older adults and the nurse who provided guidance on fall risks. Thus, it is assumed that these elements of communication have contributed to greater motivation, understanding of information and improvement of the outcome studied.

This idea is sustained by considering that communication influences people’s behavior and that the social representations of older adults about care in health services are associated with professionals’ respect, attention and education and are related to conversation, explanation and interest in helping them^(20–21)^. This influence was observed in an American study, in which older adults who participated in an educational program, with guidance from professionals on falling risks, changed behaviors: 67% started to practice physical exercise, 95.8% identified risks of falling at home and 87.3% adapted their home^(22)^.

Regarding the role of nurses as educators in elder health, their actions are necessary to motivate this population to perceive the risks of falls and the need for self-care and behavioral changes. The adoption of guiding instruments, such as the SOP, used in this study, can contribute to this process. Thus, it is urgent that such intervention be practiced and perfected by nurses, especially in the actions developed in the context of PHC to increase older adults’ perception about fall risks.

Given the strategies available to nurses to provide the process of health education for older adults in the community, the video is presented as a tool that favors breaking the paradigm of technological exclusion of this population. Thus, the use of technologies already widely used by the young is emerging for this public.

Regarding the use of educational video, it was observed, in the intragroup analysis, an increase in the mean score of 2.5 points. Other studies, identified in an integrative literature review, showed the improvement of different outcomes related to falls in older adults after the use of this type of technology. In Australia and the United States, the use of educational video promoted improved self-perception, identification and reduction of fall risks. Moreover, in the Netherlands, it was effective in improving communication techniques for deaf older adults, and in Japan, it was effective in increasing decision-making and changing preferences for life-support treatment^(11)^. These results show that this technological resource is presented as a tool that can favor the multiplication of information on fall risks to this population.

The multimedia elements used in the educational video may have contributed to improving the outcome of this study. The Cognitive Theory of Multimedia Learning, adopted in the production of the video used in this essay, is based on the ­potential in audiovisual resources to improve learning, since memory processing does not occur in a single way, but the sum of various stimuli (visual, auditory), and states that the ­construction of knowledge occurs when there is integration of prior knowledge with new content. Furthermore, this integration occurs more effectively through simultaneous stimulation with visual and verbal content. Therefore, the theory points out 12 principles that guide the multimedia planning and elaboration: coherence; signaling; redundancy; spatial contiguity; temporal contiguity; segmentation; pre-training; modality; multimedia; personalization; voice; and image^(23)^. Thus, it is believed that pictorial exposure allowed to expand the understanding of the information that was narrated, in a way that promoted an increase in the perception of falling risks.

However, it should be considered that people learn in different ways and this pluralistic essence can reflect in the results of educational interventions. Changes in attitudes of older adults are related to behaviors and life routine so that they directly influence the way they deal with health learning ­processes^(24)^. Thus, it is possible that participants’ specific ­characteristics and preferences influence the results observed in educational interventions. Thus, the diversification of teaching strategies for older adults in the community makes it possible to achieve different modes of learning. In addition to this, it contributes to assisting the public in different realities. For instance, the video can assist cases in which there is no feasibility of performing verbal nursing guidelines or in places where difficult access compromises the continuous presence of health professionals.

In the comparison of the correct answers between groups in the post-test, in each item of the questionnaire, there was a statistically significant difference in only three items, referring to the perception that most falls result in no effect (item 3), sleeping pills increase the chance of falling (item 18) and older women are more likely to fall (item 29). In these three items, the most correct answers were recorded in CG. In the others, the groups had similar effectiveness. This shows that, in this study, verbal nursing guidelines were superior to the use of educational video to generate a significant increase, specifically in the aforementioned items.

It is assumed, therefore, that this difference between groups is due to the brief presentation to elucidate this information in the video, so that verbal guidance has enabled greater prominence and clarity to these items. This finding may also suggest that, since the sample size was calculated to provide appropriate statistical power to detect differences in the primary outcome, it is likely that the statistical power achieved was not sufficient to detect greater differences in the secondary outcome. Possibly, a study with a larger sample size would be necessary to detect greater differences in the perception of each risk investigated by the questionnaire.

This study is a pioneer in Brazil, since it fills a knowledge gap and has important implications for nurses’ practice in accessible and low-cost interventions. Although comparing the two interventions, the effect size was small, there was an increase in older adults’ perception about fall risk in both groups. Based on these findings, shared decision-making to select the best health education strategy should be encouraged, considering the target audience’s preferences and perspectives, as well as nurses’ available resources and skills. It is emphasized, therefore, the importance of this professional to program, structure and value the therapeutic moment built during health education actions with older adults. Thus, it is important to emphasize the importance of investing in the permanent education for nurses to prevent falls in older adults in the community, with a view to promoting the adoption of educational strategies based on robust scientific evidence, such as those produced in this study. Access to the educational video, based on the wide dissemination by public, private or non-governmental institutions interested in the theme, as well as the good planning of verbal nursing guidelines, can contribute to reduce the prevalence of falls in this public.

Future studies are needed to assess the combined effect of using an educational video and verbal nursing guidance in older adults in the community. The mechanisms by which there is an increase in older adults’ perception about falling risks after educational interventions, as well as their predictors, need to be further investigated.

The limitations of this study include only an assessment of the perception of falling risks after the interventions, in the follow-up period of 30 days, since the assessment in more than one moment and with a longer time interval could elucidate different results. Also, due to the nature of the interventions, it was not possible to blind the team members who conducted them and the participants. Finally, the assessment of the effect of the interventions occurred in older adults in the community, Unified Health System (*Sistema Único de Saúde*) users, which may differ from the results obtained in interventions with institutionalized older adults or who are users of private health services

## CONCLUSION

The use of educational video and verbal nursing guidelines increased older adults’ perception about falling risks, with a statistical difference that points out better results in the group that received verbal guidelines. However, the effect size was small to be considered clinically important.

## ASSOCIATE EDITOR

Marcia Regina Martins Alvarenga
